# Supplementary material for: Stacking Model‐Based Classifiers for Dealing With Multiple Sets of Noisy Labels
Source: Biom J. 2025 Mar 12;67(2):e70042. doi: 10.1002/bimj.70042 (PMC11898607; doi:10.1002/bimj.70042)
Supplement: Supplementary file 1 — Supporting Information [file BIMJ-67-e70042-s001.zip › density-based-ensemble-model-main/competitors_real_data/netlab-master/netlab-master/help/gp.htm]

Netlab Reference Manual gp


# gp

## Purpose

Create a Gaussian Process.

## Synopsis

```
net = gp(nin, covarfn)
net = gp(nin, covarfn, prior)
```

## Description

`net = gp(nin, covarfn)` takes the number of inputs `nin`
for a Gaussian Process model with a single output, together
with a string `covarfn` which specifies the type of the covariance function,
and returns a data structure `net`. The parameters are set to zero.

The fields in `net` are

```
  type = 'gp'
  nin = number of inputs
  nout = number of outputs: always 1
  nwts = total number of weights and covariance function parameters
  bias = logarithm of constant offset in covariance function
  noise = logarithm of output noise variance
  inweights = logarithm of inverse length scale for each input 
  covarfn = string describing the covariance function:
      'sqexp'
      'ratquad'
  fpar = covariance function specific parameters (1 for squared exponential,
   2 for rational quadratic)
  trin = training input data (initially empty)
  trtargets = training target data (initially empty)
```

`net = gp(nin, covarfn, prior)` sets a Gaussian prior on the
parameters of the model. `prior` must contain the fields
`pr_mean` and `pr_variance`. If `pr_mean` is a scalar,
then the Gaussian is assumed to be isotropic and the additional fields
`net.pr_mean` and `pr_variance` are set. Otherwise,
the Gaussian prior has a mean
defined by a column vector of parameters `prior.pr_mean` and
covariance defined by a column vector of parameters `prior.pr_variance`.
Each element of `prmean` corresponds to a separate group of parameters, which
need not be mutually exclusive. The membership of the groups is defined
by the matrix `prior.index` in which the columns correspond to the elements of
`prmean`. Each column has one element for each weight in the matrix,
in the order defined by the function `gppak`, and each element
is 1 or 0 according to whether the parameter is a member of the
corresponding group or not. The additional field `net.index` is set
in this case.

## See Also

`gppak`, `gpunpak`, `gpfwd`, `gperr`, `gpcovar`, `gpgrad`

---

**Pages:**
Index


---

Copyright (c) Ian T Nabney (1996-9)
